# The genus *Orientoglypta* Kuslitzky, 1973 (Ichneumonidae, Banchinae) from China with descriptions of four new species

**DOI:** 10.3897/BDJ.13.e174852

**Published:** 2025-12-19

**Authors:** Jiabao Gong, Jiachen Zhu, Pu Tang, Qiong Wu, Cornelis van Achterberg, Xuexin Chen

**Affiliations:** 1 State Key Laboratory of Rice Biology, Institute of Insect Sciences, Zhejiang University, Hangzhou, China State Key Laboratory of Rice Biology, Institute of Insect Sciences, Zhejiang University Hangzhou China; 2 Zhejiang Provincial Key Lab of Biology of Crop Pathogens and Insects, Institute of Insect Sciences, Zhejiang University, Hangzhou, China Zhejiang Provincial Key Lab of Biology of Crop Pathogens and Insects, Institute of Insect Sciences, Zhejiang University Hangzhou China; 3 Zhejiang Provincial Key Laboratory of Biology and Ecological Regulation of Crop Pathogens and Insects, Zhejiang University, Hangzhou, China Zhejiang Provincial Key Laboratory of Biology and Ecological Regulation of Crop Pathogens and Insects, Zhejiang University Hangzhou China; 4 Institute of Insect Sciences, College of Agriculture and Biotechnology, Zhejiang University, Hangzhou, China Institute of Insect Sciences, College of Agriculture and Biotechnology, Zhejiang University Hangzhou China

**Keywords:** Banchinae, Glyptini, *
Orientoglypta
*, China, new species, taxonomy, key

## Abstract

**Background:**

The genus *Orientoglypta* Kuslitzky, 1973 (Hymenoptera, Ichneumonide, Banchinae) is a small taxon of ichneumonid wasps, comprising only three described species worldwide. Currently, there are no documented host records for any species. Prior to this study, *Orientoglypta* was poorly documented in China, with only a single species, *O. lota*, previously reported from Taiwan.

**New information:**

Here, we report for the first time the presence of *Orientoglypta* in mainland China, reporting four new species and one known species: *Orientoglypta lota* (Chiu, 1965), *O. laevis* Gong & Chen, sp. nov., *O. absenta* Gong & Chen, sp. nov., *O. densipunctata* Gong & Chen, sp. nov. and *O. aureocincta* Gong & Chen, sp. nov. Additionally, a key to the world species of *Orientoglypta* is provided.

## Introduction

The subfamily Banchinae (Hymenoptera, Ichneumonidae) is a highly diverse group, which contains three tribes, Atrophini, Banchini and Glyptini and more than 1800 described species worldwide (Chandra and Gupta 1977, Yu and Horstmann 1997, Broad 2014, Yu et al. 2016). Banchines are known as koinobiont endoparasitoids of lepidopterous larvae. For example, the tribe Glyptini are mainly parastoids of Tortricidae (Slovak 1987, Lu et al. 2005, Sheng and Sun 2010, Brock 2017, Sheng et al. 2018). However, the study of Banchinae remains relatively limited in China, where only 157 species have been recorded to date (Chiu 1965, He et al. 1996, He et al. 2004, Sheng and Sun 2010, Sheng et al. 2014). *Orientoglypta* currently lacks any documented host records. Moreover, host information for most Banchinae remains highly fragmentary and often unreliable even when published. Based on the number of specimens collected and its distribution range, *Orientoglypta lota* is the dominant species within the genus. Nevertheless, host records for this species remain unknown. Therefore, future field collections should prioritise careful collection and rearing of host specimens to facilitate the resolution of these taxonomic challenges. Consequently, further taxonomic research on this subfamily is necessary.

The genus *Orientoglypta* Kuslitzky, 1973 is a small taxon of ichneumonid wasps of the tribe Glyptini, subfamily Banchinae, currently containing only three species worldwide, viz. *O. lota*, *O. watanabei* and *O. aditiae* from the Eastern Palaearctic and Oriental Regions (Momoi 1963, Momoi 1965, Kuslitzky 1973, Gupta 2002). *Orientoglypta* was treated as a subgenus of the genus *Zygoglypta* Momoi, 1965 by Kuslitzky (1973) and he included a single Palaearctic species Z. (O.) watanabei Momoi. Gupta (2002) transferred many members previously classified into the genus *Zygoglypta* Momoi, 1965, including *O. watanabei*, to the genus *Glyptopimpla*. Watanabe (2017) summarised the morphological evidence for the recognition of two genera, defining *Glyptopimpla* and *Orientoglypta* as follows: 1) smooth area on frons is large in *Glyptopimpla*, but small in *Orientoglypta*; 2) the intersection of the occipital and hypostomal carinae is distant from mandibular base in *Glyptopimpla*, but close to mandibular base in *Orientoglypta*; 3) areolet present, vein 3rs-m of fore wing usually long in *Glyptopimpla*, but short in *Orientoglypta*. Watanabe (2017) transferred three species from *Glyptopimpla* to *Orientoglypta*: *O. aditiae* (Gupta, 2002), *O. lota* (Chiu, 1965) and *O. watanabei* (Momoi, 1963).

In this paper, we describe and illustrate four new species, *O. laevis* sp. nov., *O. absenta* sp. nov., *O. densipunctata* sp. nov. and *O. aureocincta* sp. nov., as well as one known species, *O. lota* and we provide a key to all known species of *Orientoglypta* worldwide.

## Materials and methods

The specimens examined in this study were collected by using sweep nets, Malaise traps and yellow pan traps. All of the specimens examined in this study are deposited in the Parasitic Hymenoptera Collection of Zhejiang University, Hangzhou (ZJUH, China).

The terminology and measurements used follow Broad et al. (2018). The following abbreviations are used in the morphological description: basal width of mandible (BWM); length of malar space (MSL); segment of antennal flagellum (F); ocello-ocular line (OOL), this being measured by the minimum distance between left lateral ocellus and eye; postocellar line (POL), this being measured by the minimum distance between both lateral ocelli; ocellus diameter (OD), this being measured by the length of a lateral ocellus. All descriptions and measurements were determined under a ZEISS Stemi 305 microscope and all the figures were made using a digital KEYENCE VHX-7000C microscope (Keyence, Osaka, Japan). The type specimens were deposited in the Parasitic Hymenoptera Collection of Zhejiang University (Hangzhou, China, ZJUH). The photos were partly processed using Adobe Photoshop 2023, mainly by cropping and modification of the background.

## Taxon treatments

### Orientoglypta

Kuslitzky, 1973

45CEFA55-08B6-54BD-9592-7164C9F8866A

Zygoglypta (Orientoglypta) Kuslitzky, 1973: 895; Yu and Horstmann 1997: 108.
Type species: *Glypta watanabei* Momoi, 1963, by original designation.
Orientoglypta : *Watanabe 2017*: 52–57.

#### Diagnosis

Occipital carina complete dorsally (but usually weak medially). Frons largely punctate, except for small area above antennal sockets. Ventral margin of clypeus without a median notch. Epomia strong and relatively long. Areolet present, vein 3rs-m of fore wing usually short. Hind wing nervellus intercepted at lower part (Townes 1970). Dorso-lateral carina and latero-median carina of metasomal tergite 1 usually complete (Watanabe 2017). Scutellum black. Antenna almost as long as body. Fore tibial spur shorter than half length of fore basitarsus.

#### Distribution

Eastern Palaearctic and Oriental Regions.

#### Biology

Unknown.

### Orientoglypta lota

(Chiu, 1965)

116EFCA5-60E4-5EBB-B30D-C80CAA9F28A4

Teleutaea lota Chiu, 1965: 208.
Synonymy: *Teleutaea townesi* Chiu, 1965: 208–209; Townes 1984: 202; Yu and Horstmann 1997: 108.
Zygoglypta lota : Townes 1970: 11; Yu and Horstmann 1997, 108.
Zygoglypta (Zygoglypta) lota :Yu and Horstmann 1997: 108.
Glyptopimpla lota : Gupta 2002: 227–228.
Orientoglypta lota : Watanabe 2017: 52–56.

#### Materials

- **Type status:** Other material. **Occurrence:** catalogNumber: No.20058289(ZJUH); recordedBy: Yiping Wang; sex: female; occurrenceID: 0F83A674-F6DD-589B-B78C-21E5FEE5EE8D; **Location:** country: China; stateProvince: Guangxi; locality: Jiuwandashan Nature Reserve; **Event:** verbatimEventDate: 20.Ⅶ.2003; **Record Level:** basisOfRecord: PreservedSpecimen
- **Type status:** Other material. **Occurrence:** catalogNumber: Nos.994018, 994077, 994020, 994076, 994053, 994043, 994058, 994014, 994013, 994015, 994078, 994021, 994064, 994052, 994049(ZJUH); recordedBy: Mingshui Zhao; sex: 15 females; occurrenceID: AAA2E6D1-BBA2-5F99-8BEE-D52C70040CA6; **Location:** country: China; stateProvince: Zhejiang; locality: Tianmu Mountain; **Event:** verbatimEventDate: 3.Ⅷ.1998; **Record Level:** basisOfRecord: PreservedSpecimen
- **Type status:** Other material. **Occurrence:** catalogNumber: Nos.20039601, 20040355, 20040377(ZJUH); recordedBy: Xuexin Chen; sex: 2 females 1 male; occurrenceID: 10BE8B97-D128-5BEE-8C2E-6BB320E15B62; **Location:** country: China; stateProvince: Zhejiang; locality: Tianmu Mountain; **Event:** verbatimEventDate: 29.Ⅶ.2003; **Record Level:** basisOfRecord: PreservedSpecimen
- **Type status:** Other material. **Occurrence:** catalogNumber: Nos.201101300, 201101864, 201101321, 201101648, 201101678(ZJUH); recordedBy: Zhen Liu; sex: 5 males; occurrenceID: 90ED12C9-FA92-55D9-A27E-25135303ACA7; **Location:** country: China; stateProvince: Zhejiang; locality: Tianmu Mountain; **Event:** verbatimEventDate: 27.Ⅶ.2011; **Record Level:** basisOfRecord: PreservedSpecimen
- **Type status:** Other material. **Occurrence:** catalogNumber: No.202503541(ZJUH); recordedBy: Jiabao Gong; sex: male; occurrenceID: 8A91E950-5A23-5BB6-9353-387B954072D3; **Location:** country: China; stateProvince: Fujian; locality: Daiyun Mountain Nature Reserve; **Event:** verbatimEventDate: 17.Ⅳ.2025; **Record Level:** basisOfRecord: PreservedSpecimen

#### Diagnosis

**Figure 1.**
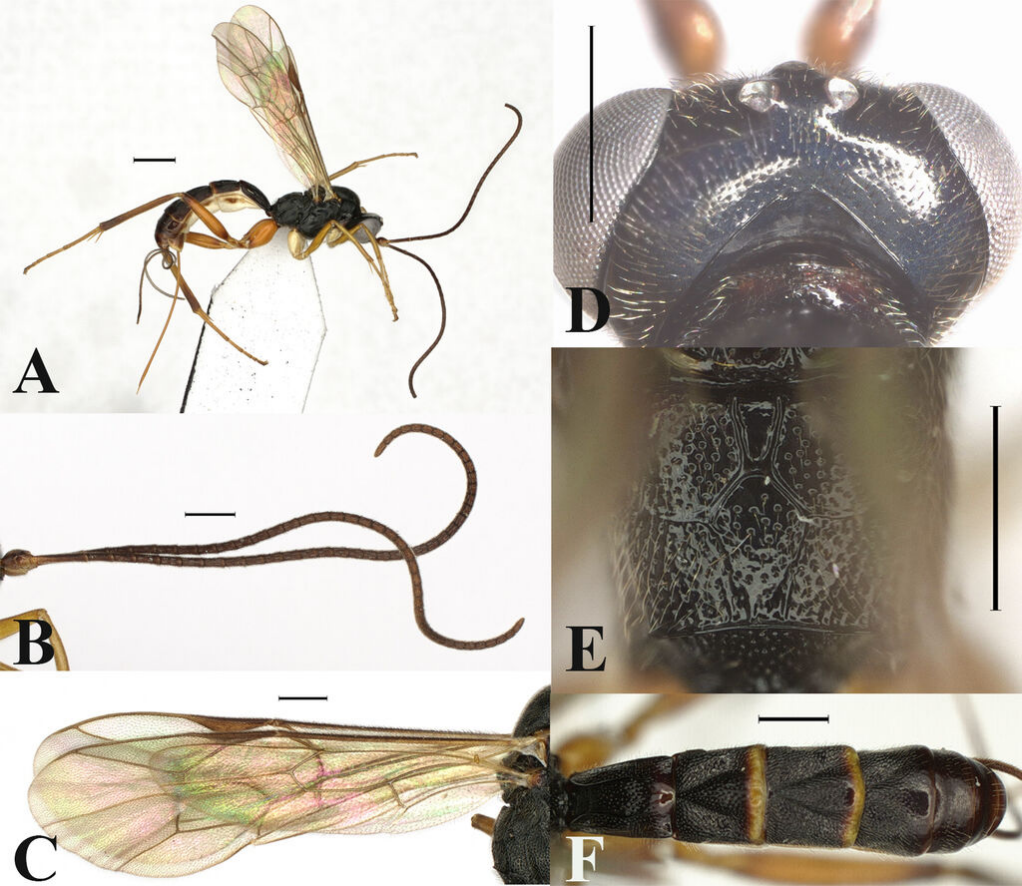
*Orientoglypta lota* (Chiu, 1965). ♀, China, Zhejiang. **A** Habitus, lateral aspect; **B** antenna; **C** wings; **D** occipital carina, dorsal aspect; **E** propodeum, dorsal aspect; **F** metasoma, dorsal aspect. Scale bars: 1 mm (A); 0.5 mm (B–F).

Dorsal part of occipital carina reverse V-shaped, strongly angled, weak in the middle (Fig. 1D). Posterior margins of metasomal tergites 2–4 with yellow narrow bands, 0.1–0.2 times as long as tergite (Fig. 1F). Area basalis of propodeum enlongated and narrowed posteriorly, smooth (Fig. 1E).

#### Distribution

China (Zhejiang, Guangxi, Fujian, Taiwan).

#### Biology

Unknown.

#### Notes

This species resembles *O. watanabei*, *O. densipunctata* and *O. aureocincta*, but it can be distinguished by the following character states: occipital carina strongly angled in dorsal part (roundly arched in dorsal part in *O. watanabei*). Its differences from *O. densipunctata* and *O. aureocincta* are described below.

### Orientoglypta laevis

Gong & Chen sp. nov.

0DAD89BD-AC60-5A8A-A2D4-5D16D3F7FE14

73F971E1-60E7-4950-BE84-B922C5935E49

#### Materials

- **Type status:** Holotype. **Occurrence:** catalogNumber: No.202503614(ZJUH); recordedBy: Tingting Zhang; sex: female; occurrenceID: FC3020ED-E7DE-5A82-A5C0-B48E78A094DD; **Location:** country: China; stateProvince: Xizang; locality: Zhamo Road of Motuo; verbatimCoordinates: 29.635N, 95.488E; **Event:** verbatimEventDate: 26.Ⅶ.2018; **Record Level:** basisOfRecord: PreservedSpecimen
- **Type status:** Paratype. **Occurrence:** catalogNumber: No.202503612(ZJUH); recordedBy: Yang Li; sex: female; occurrenceID: 4ACD5535-2D1A-5954-BEAE-6F685B8AB1B3; **Location:** country: China; stateProvince: Xizang; locality: Yigong Tea Plantation of Nyingchi; **Event:** verbatimEventDate: 5.Ⅷ.2019; **Record Level:** basisOfRecord: PreservedSpecimen
- **Type status:** Paratype. **Occurrence:** catalogNumber: No.202503613(ZJUH); occurrenceID: 6332FEB2-D14D-51AA-AF26-699C91D22D0B; **Location:** country: China; stateProvince: Xizang; locality: Tongjia village, Yigong, Nyingchi; **Event:** samplingProtocol: Yellow Pan Trap; verbatimEventDate: 6.Ⅷ.2019; **Record Level:** basisOfRecord: PreservedSpecimen

#### Description

**Female.** Body slender, its length 6.6–7.4 mm. Fore wing length 5.2–5.4 mm.

Head 0.7–0.8 times as long as wide; clypeus 0.5–0.6 times as long as wide. Anterior tentorial pit small. Face punctate, convex medially, 0.8–0.9 times as long as wide. MSL 0.7–0.8 times as long as BWM. Frons narrowly smooth above antennal sockets, punctate upper 0.7. OOL 1.3–1.5 times as long as OD; IOD 0.7–0.9 times as long as OD; interocellar area without pit. Vertex punctate; gena smooth coverd with sparse punctures. Occipital carina distinct dorsally, almost roundly arched in dorsal part, usually slightly reduced medially. Antenna with 36–37 flagellomeres. F1 2.0–2.2 times as long as F2.

**Figure 2.**
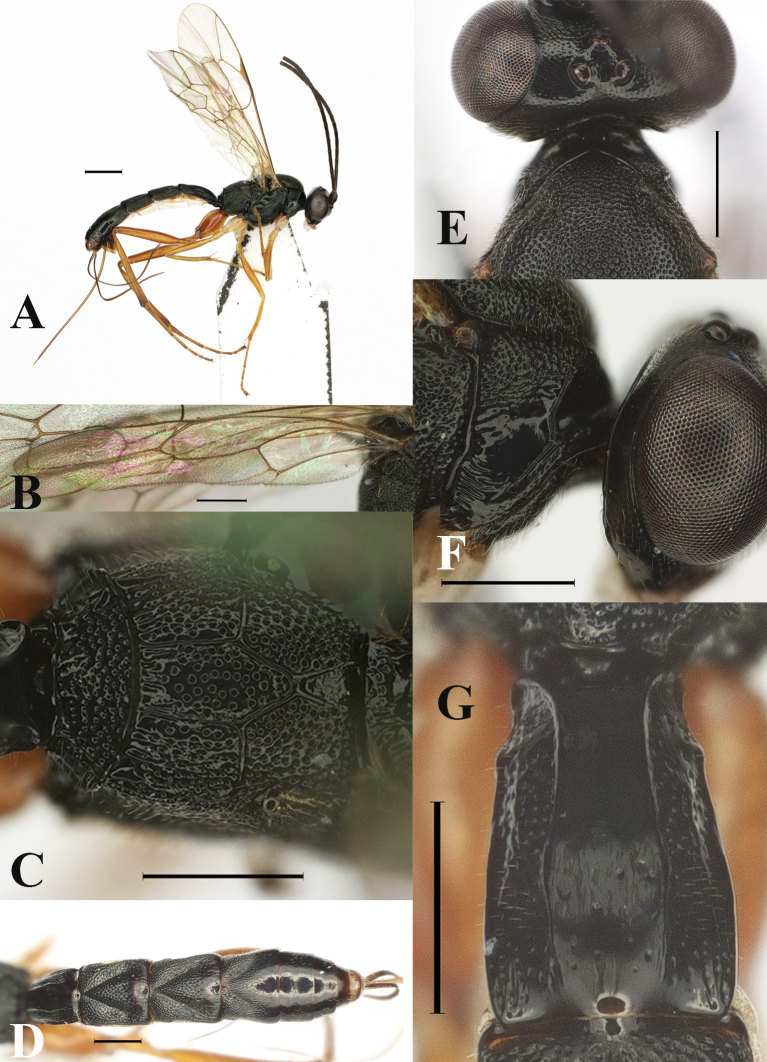
*Orientoglypta laevis* sp. nov., ♀, holotype. **A** Habitus, lateral aspect; **B** hind wing; **C** propodeum, dorsal aspect; **D** metasoma, dorsal aspect; **E** collar, dorsal aspect; **F** pronotum, lateral aspect; **G** first metasomal tergite, dorsal aspect. Scale bars: 1 mm (A); 0.5 mm (B–G).

Mesosoma punctate. Lateral area of pronotum largely smooth in ventral part (Fig. 2F). Anterior projection of submetapleural carina strongly angulate at posterior angle. Distal abscissa of vein CU of hind wing present (Fig. 2B). Posterior transverse carina, pleural carina and anterior transverse carina of propodeum complete (Fig. 2C). Area basalis of propodeum rough (Fig. 2C). Punctures on propodeal area superomedia as dense as area externa and area dentipara (Fig. 2C). Latero-median longitudinal carinae of propodeum present, its part between anterior and posterior transverse carinae of propodeum weaker than part before anterior transverse carina (Fig. 2C). Latero-longitudinal carina on propodeum partly present. Hind femur 5.7–6.2 times as long as maximum width in lateral view. Hind tibia 8.1–9.3 times as long as maximum width in lateral view. Hind basitarsus 2.1–2.3 times as long as second hind tarsal segment.

Metasoma. Metasomal tergite 1 sparsely punctate, except for smooth area between latero-median carinae (Fig. 2G). Metasomal tergites 2–4 densely punctate, except for middle part of posterior margin and posterior half of tergite 4 (Fig. 2D). Oblique grooves on metasomal tergites 2–4 moderately impressed (Fig. 2D). Metasomal tergite 1 1.8–2.0 times as long as maximum width, latero-median carina present and inner area between latero-median carinae smooth (Fig. 2G). Metasomal tergite 2 1.2 times as long as maximum width. Metasomal tergites 2 and 3 each without a pair of short latero-longitudinal keels. Ovipositor sheath 1.6 times as long as hind tibia.

Colour. Body (excluding wings and legs) black, except for: clypeus, mandible, except for dark brown tip, scape and pedicel anteriorly and subgenital plate, brown; palpi and membranous part of sternites whitish-yellow; ovipositor yellowish-brown. Wings membrane hyaline; veins, pterostigma and wing base brown. Legs yellowish-brown, except for: fore and mid coxae and trochanters whitish-yellow; apex of hind tibia dark brown.

**Male.** Unknown.

#### Diagnosis

Metasomal tergites without distinct yellow or brownish bands, whole body black (Fig. 2D). Area between latero-median carinae of tergite 1 smooth, without punctures (Fig. 2G). Pronotal collar and anterior margin of lateral area of pronotum black (Fig. 2F). Punctures on propodeum area superomedia as dense as area externa and area dentipara (Fig. 2C).

#### Etymology

Named after the smooth area between the latero-median carinae of metasomal tergite 1, ''*laevis*'' is Latin for smooth.

#### Distribution

China (Xizang).

#### Biology

Unknown.

#### Notes

This species resembles *O. aditiae* and *O. absenta*, but it can be distinguished by the following character states: latero-median carina of metasomal tergite 1 distinct and reaching posterior margin (latero-median carina of metasomal tergite 1 indistinct in *O. aditiae*); pronotum matt dorsally (pronotum shiny dorsally in *O. aditiae*); posterior transverse carina, pleural carina and anterior transverse carina of propodeum complete and distinct (propodeum with only the posterior transverse carina present in *O. aditiae*). Its differences from *O. absenta* are described below.

### Orientoglypta absenta

Gong & Chen sp. nov.

183D6877-26A3-515D-8DE2-6154B2EAEBC1

EBF7AE29-6097-4C3D-8B62-1BF0839BD409

#### Materials

- **Type status:** Holotype. **Occurrence:** catalogNumber: No.937263(ZJUH); recordedBy: Songlin Yao; sex: female; occurrenceID: DF9FE75F-7141-5A27-BBBD-A1867BB07C62; **Location:** country: China; stateProvince: Guizhou; locality: Fanjing Mountain; **Event:** verbatimEventDate: 11.Ⅶ.1983; **Record Level:** basisOfRecord: PreservedSpecimen
- **Type status:** Paratype. **Occurrence:** catalogNumber: Nos.936691, 936786, 937202, 937215, 937216, 937232(ZJUH); recordedBy: Songlin Yao and Xuexin Chen; sex: 6 females; occurrenceID: 55D83F73-C38E-5016-AF24-F94662904EDA; **Location:** country: China; stateProvince: Guizhou; locality: Fanjing Mountain; **Event:** verbatimEventDate: 11–12.Ⅶ.1983; **Record Level:** basisOfRecord: PreservedSpecimen

#### Description

**Female.** Body slender, its length 6.5–7.0 mm. Fore wing length 5.3–5.8 mm.

Head 0.7 times as long as wide; clypeus 0.6–0.7 times as long as wide. Anterior tentorial pit small. Face shallowly punctate, slightly convex medially, 0.6 times as long as wide. MSL 0.6 times as long as BWM. Frons narrowly smooth above antennal sockets, punctate upper 0.6. OOL 1.1–1.4 times as long as OD; IOD 0.8–0.9 times as long as OD; interocellar area without pit. Vertex and gena shiny and with a few punctures. Occipital carina distinct dorsally, slightly angled in dorsal part. Antenna with 35 flagellomeres. F1 1.9–2.1 times longer than F2.

**Figure 3.**
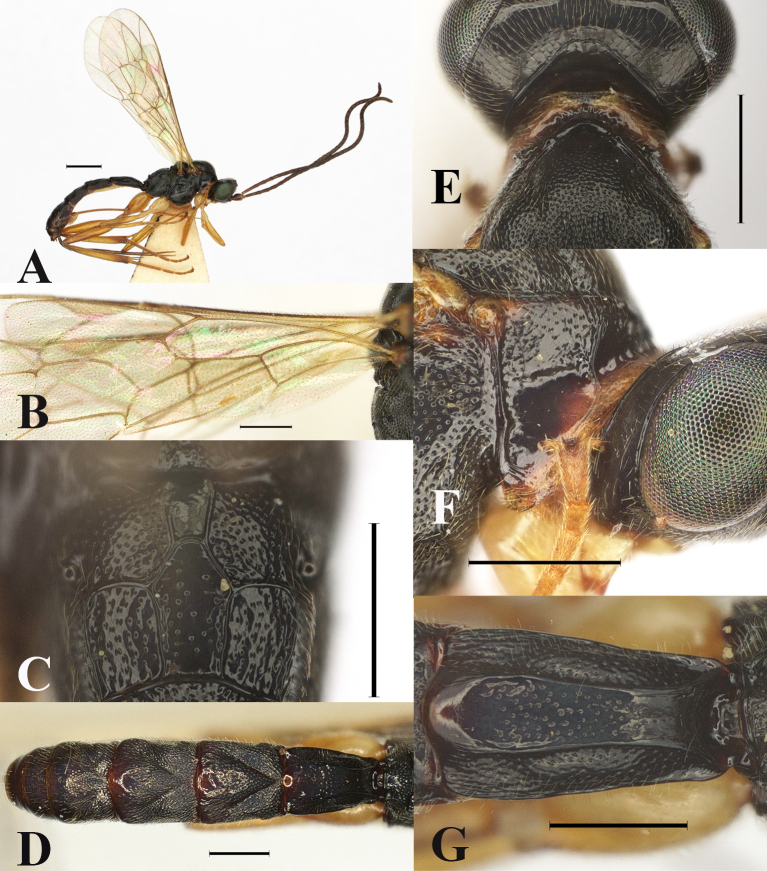
*Orientoglypta absenta* sp. nov., ♀, holotype. **A** Habitus, lateral aspect; **B** hind wing; **C** propodeum, dorsal aspect; **D** metasoma, dorsal aspect; **E** collar, dorsal aspect; **F** pronotum, lateral aspect; **G** first metasomal tergite, dorsal aspect. Scale bars: 1 mm (A); 0.5 mm (B–G).

Mesosoma punctate. Lateral area of pronotum largely smooth in ventral part (Fig. 3F). Distal abscissa of vein CU of hind wing absent (Fig. 3B). Anterior projection of submetapleural carina rounded at posterior angle. Posterior transverse carina, pleural carina and anterior transverse carina of propodeum complete (Fig. 3C). Area basalis of propodeum rough (Fig. 3C). Punctures on propodeal area superomedia sparser than on area externa and area dentipara (Fig. 3C). Latero-median longitudinal carina on propodeum present, its part between anterior and posterior transverse carinae of propodeum complete. Latero-longitudinal carina on propodeum partly present. Hind femur 4.8–5.1 times as long as maximum width in lateral view. Hind tibia 7.0–7.9 times as long as maximum width in lateral view. Hind basitarsus 1.8–1.9 times as long as second hind tarsal segment.

Metasoma. Metasomal tergite 1 sparsely punctate, except for anterior and posterior parts smooth and inner area between latero-median carinae with sparse punctures (Fig. 3G). Metasomal tergites 2–4 shallowly punctate, except for posterior margins and posterior half part of tergite 4 (Fig. 3D). Oblique grooves on metasomal tergites 2–4 moderately impressed (Fig. 3D). Metasomal tergite 1 1.6–1.8 times as long as maximum width, latero-median carina present. Metasomal tergite 2 1.0–1.1 times as long as maximum width. Metasomal tergites 2 and 3 each without a pair of short latero-longitudinal keels. Ovipositor sheath 1.8–1.9 times as long as hind tibia.

Colour. Body (excluding wings and legs) black, except for: clypeus, mandible, except tip, palpi, scape and pedicel outer spot, pronotum collar, membranous part of sternites, yellow; anterior margin of lateral area of pronotum, yellowish-brown; antenna, except for yellow outer spot of scape and pedicel, brown; small spot of pronotum before tegula, tegula and ovipositor, yellowish-brown; posterior margin of metasomal tergites medially 1–5 reddish-brown. Wings membrane hyaline; veins and pterostigma brown. Legs yellow, except for apex of hind tibia dark brown.

**Male.** Unknown.

#### Diagnosis

Distal abscissa of vein CU of hind wing absent (Fig. 3B). Metasomal tergites without distinct yellow or brownish bands, but posterior margins smooth medially and with reddish-brown metallic lustre (Fig. 3D).

#### Etymology

Named after the absent distal abscissa of vein CU of the hind wing, “*absens*” is Latin for absent.

#### Distribution

China (Guizhou).

#### Biology

Unknown.

#### Notes

This species resembles *O. laevis*, but it can be distinguished by the following character states: posterior margins of metasomal tergites smooth medially and with reddish-brown metallic lustre (metasomal tergites black, without reddish-brown metallic lustre in *O. laevis*); area between latero-median carinae of tergites 1 with sparse punctures (area between latero-median carinae of tergites 1 without punctures, smooth in *O. laevis*); pronotal collar and anterior margin of lateral area of pronotum yellowish-brown (pronotal collar and lateral area of pronotum black in *O. laevis*); punctures on propodeal area superomedia sparser than on area externa and area dentipara (punctures on propodeal area superomedia as dense as area externa and second area dentipara in *O. laevis*).

### Orientoglypta densipunctata

Gong & Chen sp. nov.

9C50E640-FB2E-54D2-A7C7-84D57089E1EA

5CC4B9F3-95E0-4CD3-9C74-307CF77219C9

#### Materials

- **Type status:** Holotype. **Occurrence:** catalogNumber: No.20004991(ZJUH); recordedBy: Jian Huang; sex: female; occurrenceID: 0C35EF31-EF0D-562A-8544-B68E5EDD1F0E; **Location:** country: China; stateProvince: Fujian; locality: Meihua Mountain of Shanghang; **Event:** verbatimEventDate: 24.Ⅶ.1988; **Record Level:** basisOfRecord: PreservedSpecimen
- **Type status:** Paratype. **Occurrence:** catalogNumber: No.887061(ZJUH); recordedBy: Junhua He; sex: female; occurrenceID: 0B5AECD4-3354-5495-8833-8DB8CB818876; **Location:** country: China; stateProvince: Fujian; locality: Meihua Mountain of Shanghang; **Event:** verbatimEventDate: 21.Ⅶ.1988; **Record Level:** basisOfRecord: PreservedSpecimen
- **Type status:** Paratype. **Occurrence:** catalogNumber: No.20007160(ZJUH); recordedBy: Changming Liu; sex: female; occurrenceID: 1C4DCAA9-B435-5B73-B1E3-4FE1365724B0; **Location:** country: China; stateProvince: Fujian; locality: Longqi Mountain of Jiangle; verbatimElevation: 1000–1500m; **Event:** verbatimEventDate: 16.Ⅶ.1991; **Record Level:** basisOfRecord: PreservedSpecimen

#### Description

**Female.** Body robust, its length 6.8–9.0 mm. Fore wing length 5.4–6.5 mm.

Head 0.6 times as long as wide; clypeus 0.6–0.7 times as long as wide. Anterior tentorial pit small. Face densely punctate, slightly convex medially, 0.6–0.7 times as long as wide. MSL 0.7 times as long as BWM. Frons narrowly smooth above antennal sockets, punctate upper 0.7. OOL 1.5 times as long as OD; IOD 0.8–1.0 times as long as OD; interocellar area without pit. Vertex punctate and gena shiny and with a few punctures. Occipital carina distinct dorsally, roundly arched in dorsal part. Antenna with 40–45 flagellomeres. F1 1.9–2.0 times longer than F2.

**Figure 4.**
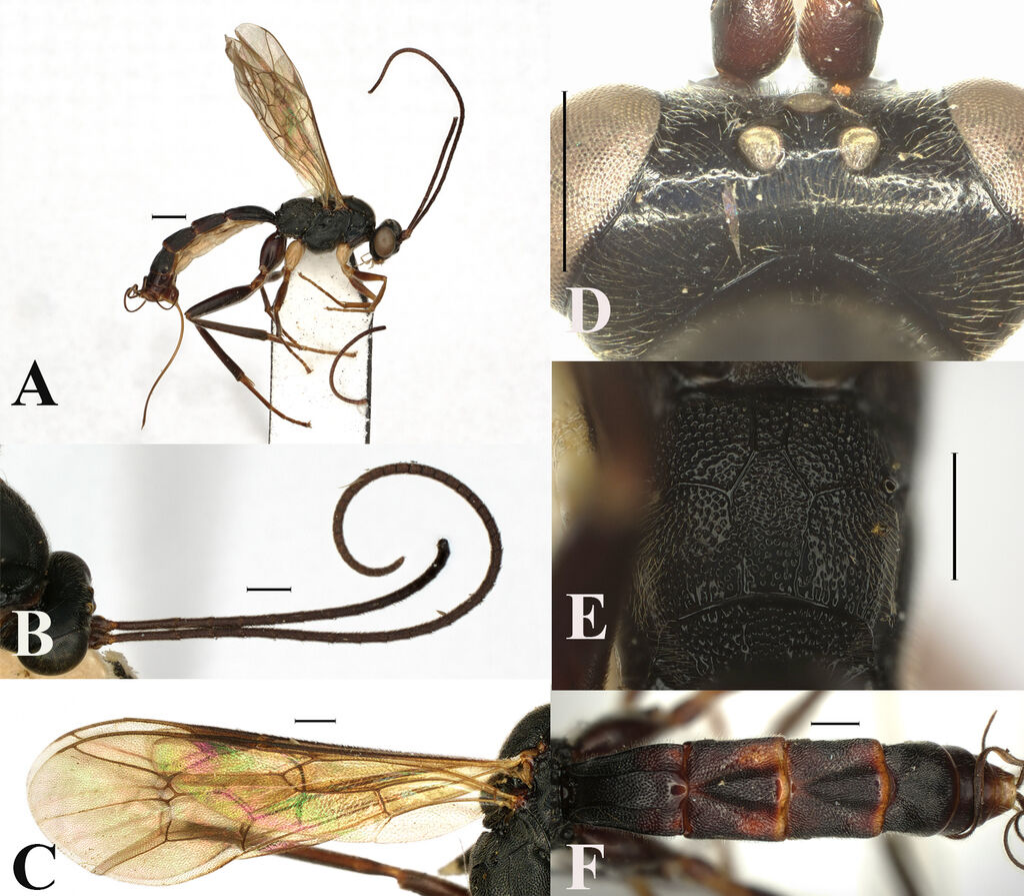
*Orientoglypta densipunctata* sp. nov., ♀, holotype. **A** Habitus, lateral aspect; **B** antenna; **C** wings; **D** occipital carina, dorsal aspect; **E** basal area of propodeum, dorsal aspect; **F** metasoma, dorsal aspect. Scale bars: 1 mm (A); 0.5 mm (B–F).

Mesosoma punctate. Lateral area of pronotum largely smooth in ventral part. Distal abscissa of vein CU of hind wing present (Fig. 4C). Anterior projection of submetapleural carina rounded at posterior angle. Posterior transverse carina, pleural carina and anterior transverse carina of propodeum complete (Fig. 4E). Area basalis of propodeum with dense punctures (Fig. 4E). Punctures on propodeal area superomedia as dense as on area externa and area dentipara (Fig. 4E). Latero-median longitudinal carina on propodeum present, its part between anterior and posterior transverse carinae of propodeum weaker than part before anterior transverse carina. Latero-longitudinal carina on propodeum partly present. Hind femur 4.6–5.0 times as long as maximum width in lateral view. Hind tibia 8.2–12.6 times as long as maximum width in lateral view. Hind basitarsus 2.1–2.4 times as long as second hind tarsal segment.

Metasoma. Metasomal tergite 1 densely punctate. Metasomal tergites 2–4 densely punctate, except for posterior narrow margins (Fig. 4F). Oblique grooves on tergites 2–3 deeply impressed (Fig. 4F). Metasomal tergite 1 1.5–1.6 times as long as maximum width, latero-median carina present and inner area between latero-median carinae with dense punctures. Metasomal tergite 2 1.0–1.1 times as long as maximum width. Metasomal tergites 2 and 3 each without a pair of short latero-longitudinal keels. Ovipositor sheath 1.3–1.9 times as long as hind tibia.

Colour. Body (excluding wings and legs) black, except for: clypeus, mandible, except tip, ovipositor, yellowish-brown; antenna, except for yellowish-brown outer spot of scape and pedicel, dark brown; membranous part of sternites, whitish-yellow; small spot of pronotum before tegula, tegula, posterior narrow margins of tergites 2–3, brown; wings hyaline; veins and pterostigma brown. Legs: hind coxa, femur and tibia, black or blackish-brown, fore and mid-coxae whitish-yellow.

**Male.** Unknown.

#### Diagnosis

Area basalis of propodeum not narrowed posteriorly, sub-rectangular, with dense punctures (Fig. 4E). Body robust (Fig. 4A). Hind femur and tibia black or blackish-brown (Fig. 4A). Oblique grooves on tergites 2–3 deeply impressed (Fig. 4F). Body almost entirely covered with dense and coarse punctures. Antenna with 40–45 flagellomeres.

#### Etymology

Named after its densely punctate body: “*densus*” is Latin for dense and “*punctum*” is Latin for puncture.

#### Distribution

China (Fujian).

#### Biology

Unknown.

#### Notes

This species resembles *O. lota*, but it can be distinguished by the following character states: hind femur and tibia black or blackish- brown (hind femur and tibia yellow, except for its brown apex and base in *O. lota*); dorsal part of occipital carina almost roundly arched (dorsal part of occipital carina reverse V-shaped, strongly angled); area basalis of propodeum with dense punctures (area basalis of propodeum smooth in *O. lota*); oblique grooves on tergites 2–3 deeply impressed (oblique grooves on tergites 2–3 moderately impressed in *O. lota*).

### Orientoglypta aureocincta

Gong & Chen sp. nov.

AF2F5163-00B3-57E1-A181-85A7439A73F2

9F94925A-1DA9-42B0-A01B-F37C0F689C4D

#### Materials

- **Type status:** Holotype. **Occurrence:** catalogNumber: No.202308692 (ZJUH).; recordedBy: Huayan Chen; sex: female; occurrenceID: 9773970E-F724-52BA-932F-6C24C6ECCB4C; **Location:** country: China; stateProvince: Ningxia; locality: Liupan Mountain; **Event:** verbatimEventDate: 13–14.Ⅶ.2009; **Record Level:** basisOfRecord: PreservedSpecimen

#### Description

**Female.** Body slender, its length 6.3 mm. Fore wing length 5.3 mm.

**Figure 5.**
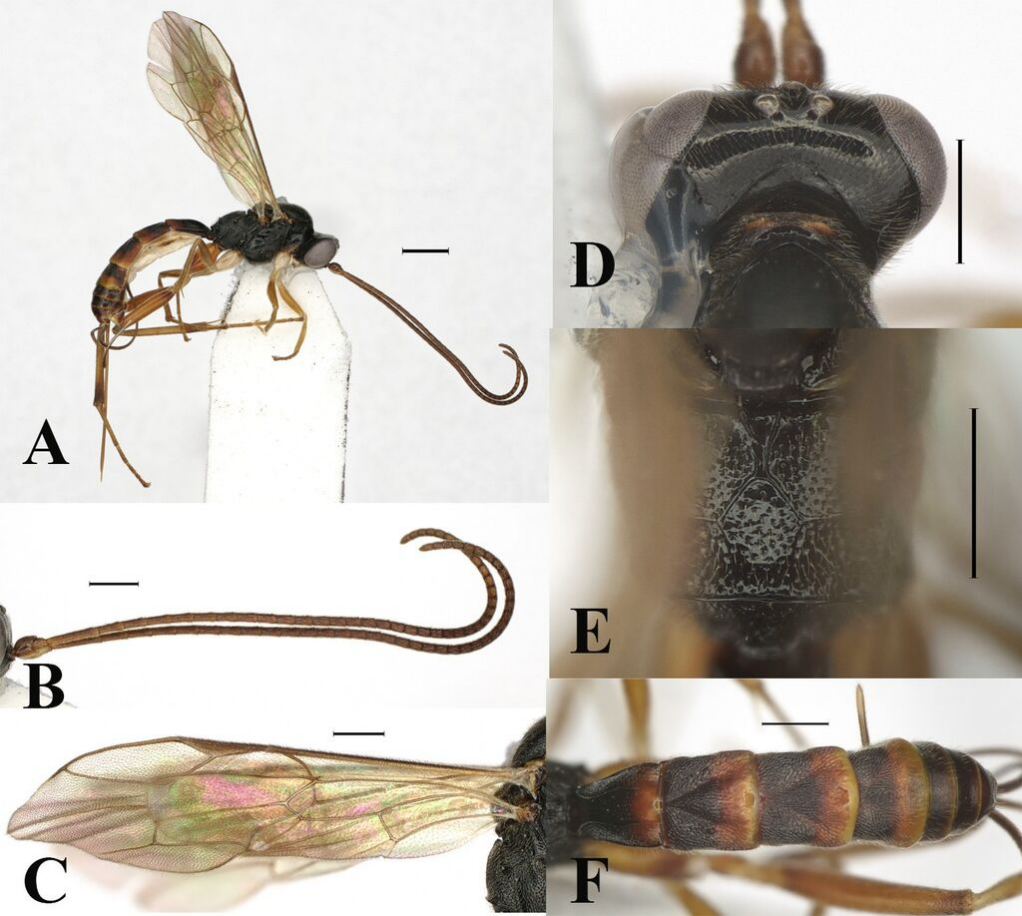
*Orientoglypta aureocincta* sp. nov., ♀, holotype. **A** Habitus, lateral aspect; **B** antenna; **C** wings; **D** occipital carina, dorsal aspect; **E** basal area of propodeum, dorsal aspect; **F** metasoma, dorsal aspect. Scale bars: 1 mm (A); 0.5 mm (B–F).

Head 0.6 times as long as wide; clypeus 0.6 times as long as wide. Anterior tentorial pit small. Face shallowly punctate, slightly convex medially, 0.7 times as long as wide. MSL 0.6 times as long as BWM. Frons narrowly smooth above antennal sockets, punctate upper 0.8. OOL 1.5 times as long as OD; IOD 0.86 times as long as OD; interocellar area without pit. Vertex and gena smooth cover with very sparse punctures. Occipital carina distinct dorsally, almost roundly arched in dorsal part (Fig. 5D). Antenna with 39 flagellomeres (Fig. 5B). F1 2.0 times as long as F2.

Mesosoma punctate. Lateral area of pronotum largely smooth in ventral part. Anterior projection of submetapleural carina rounded at posterior angle. Distal abscissa of vein CU of hind wing present (Fig. 5C). Posterior transverse carina, pleural carina and anterior transverse carina of propodeum complete (Fig. 5E). Area basalis of propodeum funnel-shaped, smooth (Fig. 5E). Punctures on propodeal area superomedia as dense as on area externa and area dentipara (Fig. 5E). Latero-median longitudinal carina on propodeum present, its part between anterior and posterior transverse carinae of propodeum weaker than part before anterior transverse carina (Fig. 5E). Latero-longitudinal carina on propodeum partly present. Hind femur 5.3 times as long as maximum width in lateral view. Hind tibia 10.0 times as long as maximum width in lateral view. Hind basitarsus 2.3 times as long as second hind tarsal segment.

Metasoma. Metasomal tergite 1 densely punctate, except for anterior and posterior parts. Metasomal tergites 2–4 densely punctate, except narrow posterior margins. Oblique grooves on tergites 2–4 moderately impressed (Fig. 5F). Metasomal tergite 1 1.6 times as long as maximum width, latero-median carina present. Metasomal tergite 2 about as long as its maximum width. Metasomal tergites 1 and 2 each without a pair of short latero-longitudinal keels. Ovipositor sheath 1.6 times as long as hind tibia.

Colour. Body (excluding wings and legs) black, except for: clypeus, mandible, except tip, palpi, scape and pedicel outer spot, whitish-yellow; antenna, except for yellow area of scape and pedicel, brown; small spot of pronotum before tegula, tegula, anterior margin of lateral area of pronotum, brown; apical bands of tergites 1–6 (tergites 1–4 broad, tergites 5 and 6 very narrow), yellowish-brown; membranous part of sternites, posterior margin of subgenital plate, whitish-yellow; remainder of sternites, including subgenital plate, brown; ovipositor yellowish-brown. Wings hyaline; veins and pterostigma brown. Legs yellowish-brown, except for: fore and mid-coxae and trochanters, whitish-yellow, hind trochanter and trochantellus and apex of hind tibia, dark brown.

**Male.** Unknown.

#### Diagnosis

Metasomal tergites 1–4 with broad yellow bands on posterior margins; bands on tergites 2–4 0.3–0.5× length of tergite (Fig. 5F). Band on tergite 4 broadest, occupying almost half of its length (Fig. 5F). Area basalis of propodeum funnel-shaped (Fig. 5E).

#### Etymology

Named after the golden and very broad bands on its anterior tergites; “*aurea*” is Latin for golden and “*cincta*” is Latin for band.

#### Distribution

China (Ningxia).

#### Biology

Unknown.

#### Notes

This species resembles *O. lota*, but it can be distinguished by the following character states: posterior margin bands of metasomal tergites 2–3 broad, 0.3–0.5× length of tergites (bands on metasomal tergites 2–3 posterior margin narrow, 0.1–0.2× length of tergites in *O. lota*); dorsal part of occipital carina almost roundly arched (dorsal part of occipital carina reverse V-shaped, strongly angled in *O. lota*); metasomal tergites 1 and 4 with distinct posterior margin bands (metasomal tergites 1 and 4 each without distinct posterior margin bands or band of metasomal tergite 1 obsolete in *O. lota*).

## Identification Keys

### Key to world species of the genus *Orientoglypta*

**Table:** 

1	Metasomal tergites without distinct yellow or brownish bands (Fig. 2D and Fig. 3D).	2
–	Metasomal tergites with distinct yellow or brownish bands (Fig. 1F, Fig. 4F and Fig. 5F).	4
2	Only posterior transverse carina of propodeum strong and distinct. MSL equal to BWM. Pronotum shiny dorsally. Face medially raised and rugulose.	*O. aditiae* Gupta, 2002
–	Propodeum with strong and distinct carinae in addition to posterior transverse carina. MSL shorter than BWM. Pronotum matt dorsally. Face medially raised, but not rugulose.	3
3	Distal abscissa of vein CU of hind wing is present (Fig. 1C, Fig. 2B, Fig. 4C and Fig. 5C). Metasomal tergites entirely black. Area between latero-median carinae of tergite 1 smooth, without punctures (Fig. 2G). Punctures on propodeal area superomedia as dense as area externa and area dentipara (Fig. 2C). Pronotal collar and anterior margin of lateral area of pronotum black (Fig. 2E and Fig. 2F).	*O. laevis* sp. nov.
–	Distal abscissa of vein CU of hind wing is absent (Fig. 3B). Posterior margin of metasomal tergites reddish-brown with metallic lustre (Fig. 3D). Area between latero-median carinae of tergite 1 with sparse punctures (Fig. 3G). Punctures on propodeal area superomedia sparser than area externa and area dentipara (Fig. 3C). Pronotal collar and anterior margin of lateral area of pronotum yellowish-brown (Fig. 3E and Fig. 3F)	*O. absenta* sp. nov.
4	Area basalis of propodeum not narrowed posteriorly, sub-rectangular and with dense punctures (Fig. 4E). Oblique grooves on tergites 2–3 deeply impressed (Fig. 4F). Antenna usually with not less than 40 flagellomeres. Hind coxa, femur and tibia black or blackish-brown (Fig. 4A).	*O. densipunctata* sp. nov.
–	Area basalis of propodeum narrowed posteriorly, smooth or with sparse punctures (Fig. 1E and Fig. 5E). Oblique grooves on tergites 2–3 moderately impressed (Fig. 1F and Fig. 5F). Antenna usually with less than 40 flagellomeres. Hind coxa, femur and tibia yellowish-brown (except for its dark brown apex and base) (Fig. 1A and Fig. 5A).	5
5	Posterior margins of metasomal tergites 2–3 with broad bands, 0.3–0.5 times as long as tergite and metasomal tergites 1, 4 with distinct posterior bands (Fig. 5F).	*O. aureocincta* sp. nov.
–	Posterior margins of metasomal tergites 2–3 with narrow bands, 0.1–0.2 times as long as tergite and metasomal tergites 1, 4 without distinct bands or only tergite 1 with an obsolete band (Fig. 1F).	6
6	Dorsal part of occipital carina reverse V-shaped, strongly angled (Fig. 1D). MSL 0.4 times as long as BWM in male.	*O. lota* (Chiu, 1965)
–	Dorsal part of occipital carina roundly arched. MSL 0.6–0.7 times as long as BWM in male.	*O. watanabei* (Momoi, 1963)

## Supplementary Material

XML Treatment for Orientoglypta

XML Treatment for Orientoglypta lota

XML Treatment for Orientoglypta laevis

XML Treatment for Orientoglypta absenta

XML Treatment for Orientoglypta densipunctata

XML Treatment for Orientoglypta aureocincta
